# The Multifaceted Origin of Taurine Cattle Reflected by the Mitochondrial Genome

**DOI:** 10.1371/journal.pone.0005753

**Published:** 2009-06-01

**Authors:** Alessandro Achilli, Silvia Bonfiglio, Anna Olivieri, Arianna Malusà, Maria Pala, Baharak Hooshiar Kashani, Ugo A. Perego, Paolo Ajmone-Marsan, Luigi Liotta, Ornella Semino, Hans-Jürgen Bandelt, Luca Ferretti, Antonio Torroni

**Affiliations:** 1 Dipartimento di Genetica e Microbiologia, Università di Pavia, Pavia, Italy; 2 Dipartimento di Biologia Cellulare e Ambientale, Università di Perugia, Perugia, Italy; 3 Sorenson Molecular Genealogy Foundation, Salt Lake City, Utah, United States of America; 4 Istituto di Zootecnica, Università Cattolica del Sacro Cuore, Piacenza, Italy; 5 Dipartimento di Morfologia, Biochimica, Fisiologia e Produzioni Animali, Università di Messina, Messina, Italy; 6 Department of Mathematics, University of Hamburg, Hamburg, Germany; University of Glasgow, United Kingdom

## Abstract

A Neolithic domestication of taurine cattle in the Fertile Crescent from local aurochsen (*Bos primigenius*) is generally accepted, but a genetic contribution from European aurochsen has been proposed. Here we performed a survey of a large number of taurine cattle mitochondrial DNA (mtDNA) control regions from numerous European breeds confirming the overall clustering within haplogroups (T1, T2 and T3) of Near Eastern ancestry, but also identifying eight mtDNAs (1.3%) that did not fit in haplogroup T. Sequencing of the entire mitochondrial genome showed that four mtDNAs formed a novel branch (haplogroup R) which, after the deep bifurcation that gave rise to the taurine and zebuine lineages, constitutes the earliest known split in the mtDNA phylogeny of *B. primigenius*. The remaining four mtDNAs were members of the recently discovered haplogroup Q. Phylogeographic data indicate that R mtDNAs were derived from female European aurochsen, possibly in the Italian Peninsula, and sporadically included in domestic herds. In contrast, the available data suggest that Q mtDNAs and T subclades were involved in the same Neolithic event of domestication in the Near East. Thus, the existence of novel (and rare) taurine haplogroups highlights a multifaceted genetic legacy from distinct *B. primigenius* populations. Taking into account that the maternally transmitted mtDNA tends to underestimate the extent of gene flow from European aurochsen, the detection of the R mtDNAs in autochthonous breeds, some of which are endangered, identifies an unexpected reservoir of genetic variation that should be carefully preserved.

## Introduction

It is generally accepted that modern cattle originated from two domestication events of aurochs (*Bos primigenius*) that took place in southwest Asia and south Asia giving rise to taurine (*Bos taurus*) and zebuine (*Bos indicus*) stocks, respectively [1]–[3]. This was followed by the spread of domesticated herds throughout the Old World accompanying human trade and migration. However, *B. primigenius* was not restricted to Asia; it was common also in North Africa and Europe as attested to by skeletal remains and Paleolithic cave paintings. Thus, distribution ranges for wild aurochsen and domestic cattle overlapped for millennia. Ancient Greek and Roman writers described the aurochs as very aggressive and Julius Caesar, who saw them in the Black Forest (Southern Germany), wrote in *Gallic War* Chapter 6.28 *“…those animals are a little below the elephant in size, and of the appearance, colour, and shape of a bull. They spare neither man nor wild beast … not even when taken very young can they be tamed.”* By the 13th century A.D., aurochsen were extremely rare and restricted to Eastern Europe, with the last recorded aurochs dying in Poland in 1627.

Cattle mitochondrial DNA (mtDNA) studies have generally focused on segments (213–910 base pairs) of the control region, revealing five major haplotype clusters in *B. taurus* (T*-T1-T2-T3-T4) and two in *B. indicus* (I1–I2) [3]–[7]. Similar studies on human mtDNA have taught us, however, that focusing exclusively on a small portion of the non-coding and fast-evolving control region can be inadequate [8].

Last year phylogeographic analyses of cattle mtDNA were brought to the highest level of molecular resolution – that of entire mitochondrial sequences [9], showing that almost all taurine mtDNAs belong to the single macro-haplogroup T, whose sequence divergence corresponds to a time estimate of only ∼16 thousand years (ky). This coalescence time indicates an extremely narrow bottleneck in the recent evolutionary history of *B. taurus*. Moreover, macro-haplogroup T shows an initial split into two sister subclades, T5 and T1'2'3. The predominant subclade T1'2'3 is formed by three major branches corresponding to the previously defined haplogroups T1, T2 and T3, whereas T4 turned out to be nested within T3. Coalescence ages and geographical distributions of the T branches support a Neolithic origin for all T mtDNAs from a Fertile Crescent aurochs population, and dismiss the possibility of other domestication foci involving T mtDNAs in different geographic areas [1], [4], [10], [11]. Indeed, multiple domestication events would have required that African, European and East Asian *B. primigenius* populations not only harboured mtDNAs belonging to T but that their T mtDNAs had remained almost identical relative to the Near Eastern counterparts during their allopatric evolution.

Yet, the analysis of mitochondrial genomes revealed that not all mitochondrial genomes from taurine breeds clustered within macro-haplogroup T [9], as three mtDNAs radiating prior to the T node in the phylogeny were also detected. One, found in an animal from Korea generically classified as “beef cattle”, harboured the control-region mutational motif for haplogroup P – the marker of the extinct aurochs of northern and central Europe [12]. The remaining two, identical to each other, were found in an endangered Italian breed (Cabannina) and belonged to a novel haplogroup, termed Q, whose split time from the QT node seemed to be compatible with an origin from European aurochsen. Overall these findings confirmed that the gene pool of taurine cattle mostly derives from the Near East, but revealed that there was also a minor genetic contribution from European aurochsen.

In the present study, we analyzed 619 mtDNAs from 26 European breeds to fully evaluate the nature and extent of gene flow from European *B. primigenius* to modern taurine cattle. The survey identified additional mtDNAs which did not fit in haplogroup T, confirming that some female European aurochsen, or perhaps their hybrid (female) offspring, were indeed included in domestic herds, thus revealing that the sources of genetic variation for taurine cattle are more heterogeneous than initially thought.

## Results

### Identification of Uncommon mtDNA Haplogroups in European Taurine Breeds

Analysis of the mtDNA control region (∼840 bp) was performed in a total of 619 samples from 26 European breeds (22 from Italy and four from other European regions). This survey allowed the identification of diagnostic control-region mutational motifs and the classification of almost all mtDNAs within known haplogroups (Table 1 and Table S1). As expected for European cattle, haplogroup T3 encompassed the vast majority of mtDNAs (89.2%) while haplogroups T1 and T2 were detected at lower frequencies (5.3% and 3.8%, respectively). None of the samples harboured the diagnostic control-region motifs of haplogroups T4 and T5. Two mtDNAs (0.3%) could not be ascribed to any of the T subclades listed above, but a sequencing survey of the T1'2'3 diagnostic marker (13005A) revealed that, similarly to one already reported sample [9], they belonged to T1'2'3. It is not unlikely that the rare T1'2'3 mtDNAs are indeed T1 or T3 mtDNAs that have reverted at their single diagnostic site (16113 and 16255, respectively), since multiple recurrent mutations at these positions are predicted by the T mtDNA tree [9].

**Table 1:** Diagnostic Control-Region Motifs for Taurine mtDNA Haplogroups.

Haplogroups	Control-region motifs^a^	No. of mtDNAs	%
T1'2'3	16255 169	2	0.3
T1	16113 16255 169	33	5.3
T2	16057C 16185 16255 169	24	3.8
T3	(169)^b^	552	89.2
T4	16042 16093 16302 169	0	-
T5	16255 163 169	0	-
P	15951 15953G 15994 16049 16051 16058 16074 16085 16122 16231 16247 16255 16264 106 166 173 190 221+C 222 249 300 301	0	-
Q	15953G 16255 169	4	0.7
R	15818 15900 15951 15953G 16057 16076 16084 16085 16121 16122 16127 16135 16137 16200+A 16231 16248 16250 16264 16301 8 106 166 173 221+C 234+T 249 296 300	4	0.7

a Diagnostic motifs of the entire control region relative to the BRS [13].

b BRS belongs to a T3 subclade (T3b) that is characterized by a transition at np 169.

Yet, there were also eight mtDNAs (1.3%) that did not cluster within any subset of T. Four mtDNAs [one Chianina (Tuscany), one Italian Red Pied (Friuli), and two Romagnola (Romagna)] harboured a control-region motif that is not very dissimilar from those observed in T mtDNAs, but is characterized by the distinguishing mutation 15953G (Table 1), thus suggesting a possible affiliation with the recently discovered haplogroup Q [9]. The other four mtDNAs [one Agerolese (Campania), two Cinisara (Sicily), and one Romagnola] were characterized by a peculiar and very divergent control-region motif (28 mutations) relative to the bovine reference sequence (BRS) [13] (Table 1).

### Phylogenetic Relationships of the Rare Haplogroups Based on the Analysis of Complete Mitochondrial Sequences

**Figure 1:**
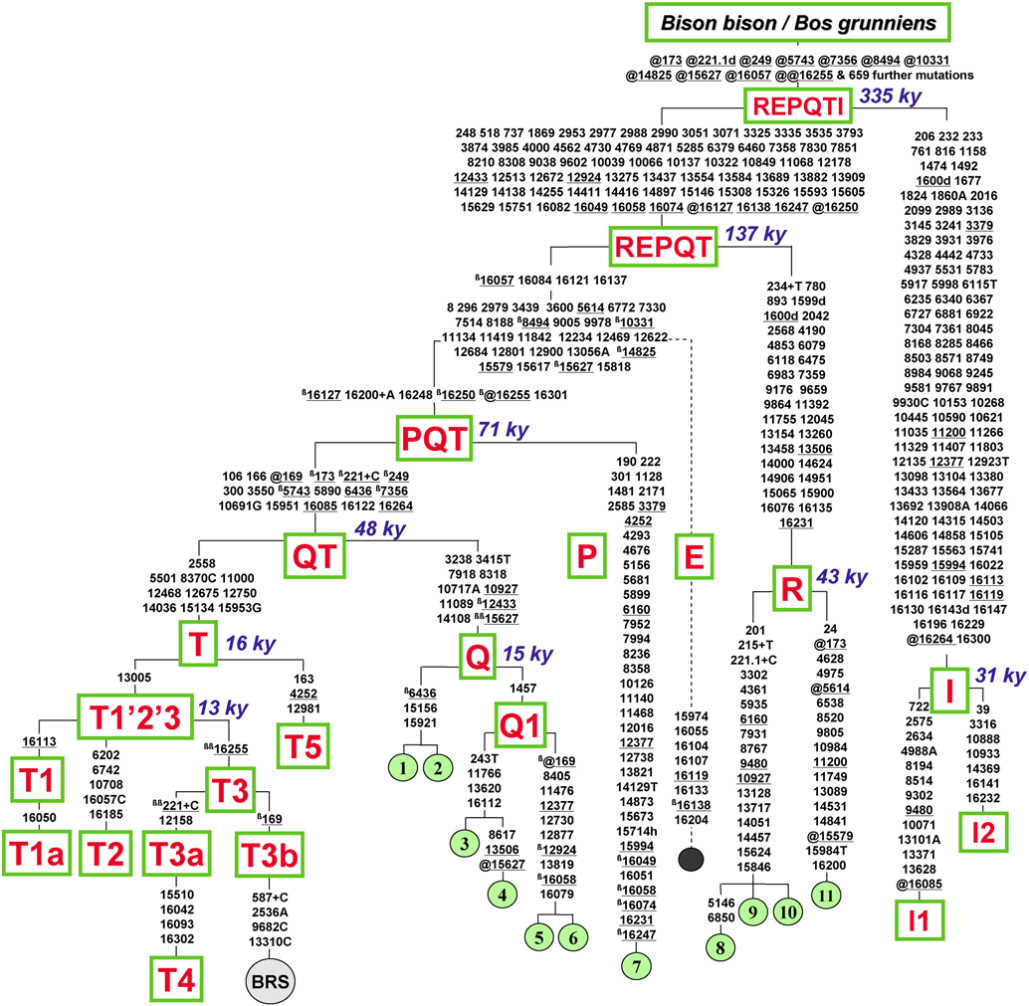
Tree of Complete MtDNA Sequences from Cattle. This tree illustrates the relationships between the common macro-haplogroups T and I and the rare mtDNAs belonging to haplogroups P, Q and R. Haplogroup E (dashed line) refers to the partial previously reported mtDNA data from a German aurochs [12], [14]. Divergence times are an average of the ML and ρ estimates reported in Table 2. BRS indicates the *Bos taurus* reference sequence (GenBank acc. no. V00654) [13]. Mutations are shown on the branches and are numbered according to the BRS; they are transitions unless a base is explicitly indicated; suffixes indicate transversions (to A, G, C, or T) or indels (+, d) and have to be read as if BRS was an artificial root. Recurrent mutations are underlined, and true back mutations with respect to evolutionary direction are prefixed with the superscript β (beta) in addition (which is thus in alternation with prefix @ on the path between the overall root and BRS). Heteroplasmy is marked with a suffix (h). Mutations explicitly listed above the REPQTI node are those reverted at least once in the branches below that node, while the additional 659 mutations are those shared between *Bison bison* and *Bos grunniens* as reported in Achilli et al. [9], except for 249@, 5743@, 7356@, 12377@, 15627@, 15994@, and 16264. Note that the reconstruction of recurrent mutations in the control region is ambiguous in a number of cases. Breeds for the samples harbouring mtDNAs belonging to haplogroups Q, P and R are as follows: Chianina (1); Romagnola (2); Italian Red Pied (3); Romagnola (4); Cabannina (5, 6); “Beef cattle”, Korea (7); Agerolese (8); Cinisara (9, 10); Romagnola (11).

The eight non-T mtDNAs were completely sequenced and their phylogenetic relationships are illustrated in Figure 1. The four postulated Q mtDNAs (sequences #1–4) confirmed their affiliation with haplogroup Q, but turned out to be quite different from the identical Q mtDNAs (sequences #5–6) previously found in the Cabannina breed [9]. Divergence values confirmed the split time of ∼48 ky for the QT node, while the estimated divergence time for the Q node was much lower (15±4 ky), thus overlapping with those observed for haplogroup T and its subclades (Table 2).

**Table 2:** Haplogroup Divergence Values and Time Estimates of Cattle mtDNA Haplogroups Obtained by Using Maximum Likelihood (ML) and ρ Statistics.

Haplogroups/Subhaplogroups	No. of mtDNAs^a^	Maximum Likelihood				ρ^b^ Statistics			
		Substitutions per site	S.E.	T (ky)^c^	±ΔT (ky)	ρ	σ	T (ky)^c^	±ΔT (ky)
REPQTI	116	0.00673	0.00047	329.5	23.2	107.240	6.676	340.2	21.2
REPQT	110	0.00276	0.00027	135.3	13.1	43.690	4.091	138.6	13.0
R	4	0.00090	0.00017	43.9	8.1	13.500	2.872	42.8	9.1
PQT	106	0.00145	0.00018	70.8	8.8	22.572	2.803	71.6	8.9
QT	105	0.00099	0.00015	48.2	7.2	15.088	2.293	47.9	7.3
Q	6	0.00031	0.00008	15.3	4.0	4.833	1.280	15.3	4.1
Q1	4	0.00027	0.00007	13.1	3.6	5.250	1.561	16.7	5.0
T	99	0.00032	0.00006	15.8	3.1	5.370	0.937	17.0	3.0
T5	4	0.00021	0.00006	10.2	3.0	3.500	1.225	11.1	3.9
T1'2'3	95	0.00024	0.00002	12.0	0.8	4.326	0.413	13.7	1.3
T1^d^	9	0.00019	0.00005	9.4	2.4	2.556	0.556	8.1	1.8
T2	16	0.00021	0.00002	10.4	0.9	4.812	0.634	15.3	2.0
T3d,e	69	0.00023	0.00002	11.3	0.9	3.779	0.362	12.0	1.1
T4^d^	7	0.00014	0.00004	6.9	2.0	2.286	0.606	7.3	1.9
I	6	0.00068	0.00014	33.3	6.7	9.062	1.896	28.7	6.0
I1	3	0.00013	0.00005	6.5	2.4	2.333	0.882	7.4	2.8
I2	3	0.00022	0.00007	10.9	3.5	3.000	1.000	9.5	3.2

a In addition to the eight new Q and R mtDNA sequences (GenBank accession numbers FJ971080 to FJ971087), these include 106 sequences previously reported by Achilli et al. [9], one by Hiendleder et al. [27], and a novel one from Mongolia belonging to haplogroup I1 (GenBank accession number FJ971088).

b Average number of base substitutions in the mtDNA coding region (between nps 364 and 15791) from the ancestral sequence type. For haplogroups REPQTI, REPQT, PQT, QT, T and I, the contribution to ρ from each subclade is weighted based on their individual standard errors.

c Estimate of the time to the most recent common ancestor of each clade, using a mutation rate estimate of 3,172 years per substitution in the whole coding region (15,428 bp) [9].

d The ML age for this haplogroup, defined only by a control-region motif, was calculated separately.

e Including T4 mtDNAs.

Data from the complete sequences of the remaining four mtDNAs (#8–11) showed that they were indeed related to each other, as already suggested by the control-region motif. They formed a novel haplogroup, termed R, which is a sister branch of superhaplogroup PQT (Figure 1), with a split time of ∼137 ky ago. The two Cinisara mtDNAs (#9–10) were identical and differed from the Agerolese mtDNA (#8) at only two coding-region positions, while the Romagnola sequence (#11) defined a separate branch, differing by a minimum of 34 mutations (Figure 1) and radiating ∼43 ky ago from its R counterparts (Table 2).

The comparison of the R sequences with partial control-region and *cyt b* sequences obtained from the skeletal remains of a ∼7 ky aurochs from Germany (lineage E) [12], [14] suggests that the aurochs E lineage falls into the same radiation as R and PQT, probably forming a sister branch of PQT rather than R. Thus, after the deep bifurcation that ∼335 ky ago gave rise to the taurine and zebuine lineages (Table 2), the separation between the new haplogroup R and the other branches (E and PQT) represents the earliest known split in the mtDNA phylogeny of *B. primigenius* (Figure 1).

## Discussion

### The Origin of the non-T mtDNAs Found in European Taurine Breeds

Our analysis of numerous cattle breeds, mainly from Italy, reveals that not all taurine mtDNAs from Europe are members of haplogroup T. As expected, T and its subclades encompass the vast majority of mtDNAs, but about 1.4% of the samples belong to two different lineages: the recently discovered haplogroup Q [9] and a novel haplogroup R. Taking into account that one mtDNA belonging to haplogroup P has also been reported [9], our data indicate that at least three non-T haplogroups (P, Q and R) are present, albeit at low frequencies, in modern European taurine breeds. The haplogroup P mtDNA matches the control-region motifs obtained from the remains of numerous northern and central European aurochsen [12], thus indicating that at least one female aurochs from Europe was able to genetically contribute to modern taurine cattle.

### Europe is the Homeland of Haplogroup R

What about the ancestral genetic and geographic sources of haplogroups Q and R? Similar to haplogroup P, could they represent the genetic legacy of the extinct European *B. primigenius*? Alternatively, could either one or both lineages have been brought to Europe from the Near East with the spread of domestic herds? Phylogeographic data provide some insights on these issues.

The separation between R and the other taurine branches represents the earliest known split in the mtDNA phylogeny of *B. primigenius* (Figure 1), pre-dating even the branching of the P lineage, which is typical of European aurochsen. Moreover, despite the R control-region motif being well defined and easily distinguishable from those of other haplogroups (Table 1), the survey of published datasets does not reveal any near-match in either modern or ancient samples [3], [12], [15], [16]. Thus, taking also into account that haplogroup R is constituted by two deep diverging branches with a split at ∼43 ky ago, the detection of R mtDNAs is best explained by sporadic events of interbreeding between European aurochsen and domestic stocks. In other words, two European female aurochsen with very different R mtDNAs, or perhaps a hybrid (female) offspring resulting from interbreeding with domestic bulls, were included in domestic herds and transmitted their mitochondrial genomes to some modern Italian breeds.

### A Near Eastern Domestication for Haplogroup Q

The phylogeographic information concerning haplogroup Q is more intriguing, and includes published data from both modern and ancient DNA. The few complete sequences belonging to Q tend to diverge in a star-like fashion, with an overall rather recent coalescence time (∼15±4 ky). Both these features are shared with the subclades of T that underwent domestication [9]. In contrast to R, haplogroup Q is phylogenetically close to haplogroup T and its control-region motif is poorly differentiated (15953G-16255-169) from the reference sequence BRS, which is a member of subclade T3b (Figure 1). Moreover, position 169 in the above motif is unstable. Despite these limitations, a search for the 15953G-16255 motif reveals some interesting matches in published datasets. Two mtDNAs with the 15953G-16255 motif have been detected in Neolithic cattle bones from two distant archeological sites, one in Germany (Goddelau, oldest Linearbandkeramic; LBK Culture) and one in Eastern Thrace, Turkey (Asagi Pinar, c. 5250–5080 cal BC) [17], [18]. With regard to modern samples, three mtDNAs, one harbouring the 15953G-16141-16255-169 motif and two sharing the 15953G-16141-16146-16255-169 motif, have been reported in local breeds of southwestern China [6], [19], while the 15953G-16140-16255-169 motif has been described in one East Anatolian Red (GenBank EF126311). Sharing the transversion at np 15953 these ancient and modern samples probably all belong to Q. However, there is also a second, less likely possibility: they could also constitute another rare, still unidentified, sister clade of either T, or Q, or QT (Figure 1), thus testifying to even more basal lines of descent in the vicinity of the QT node.

**Figure 2:**
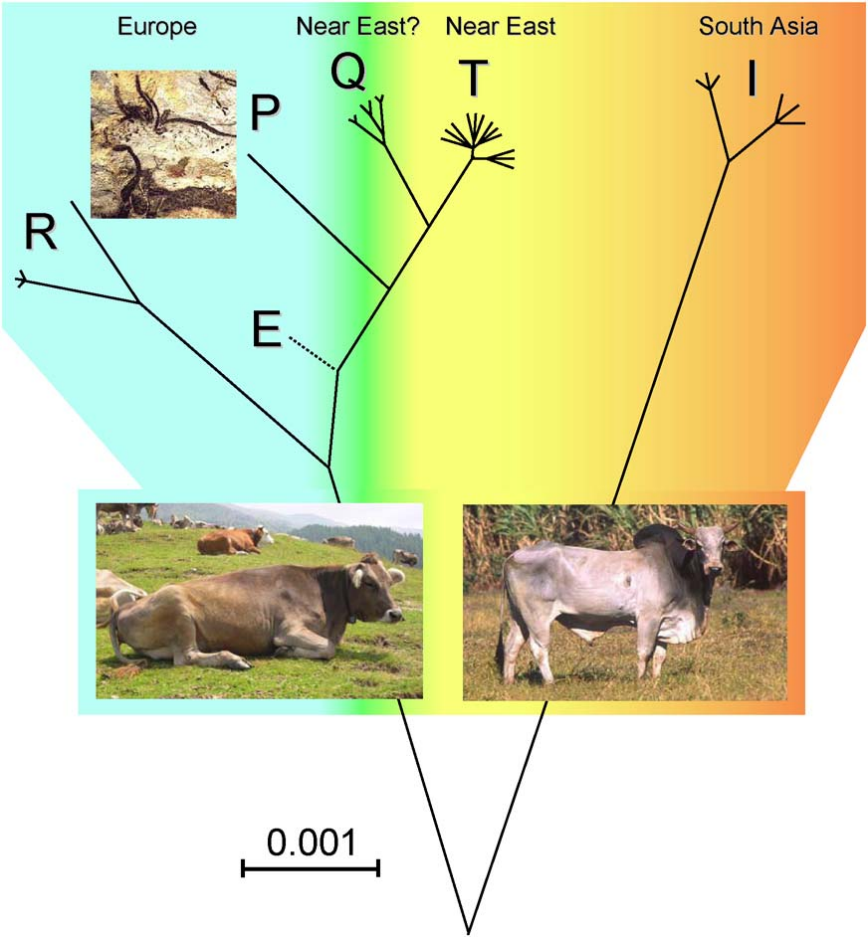
Most Parsimonious Phylogeny of Cattle MtDNA. This unrooted tree is drawn to scale using ML distances (Table 2) and includes all available P, Q and R complete mtDNA sequences, including those reported in Achilli et al. [9]. The placement of the E branch (dashed line) is based on a partial control-region and coding-region data from a German aurochs [12], [14]. The Paleolithic painting of aurochsen (*B. primigenius*) is from the Lascaux cave (southern France).

The latter possibility might be compatible with a scenario posing that modern Asian mtDNAs with 15953G were members of a novel branch distinct from Q, so that an exclusive presence of Q mtDNAs in Europe could imply that haplogroup Q, similarly to haplogroups P and R, was derived from European *B. primigenius*. However, it is more realistic to predict that the 7 ky old Neolithic samples from Europe and the modern mtDNAs from Asia are indeed members of haplogroup Q, which derived from Near Eastern aurochs populations. Therefore Q and the subclades of T could have been involved in the same domestication event in the Fertile Crescent (Figure 2). This scenario would fit well both the coalescence age of Q and its star-like structure. Moreover, the frequency and distribution of Q would parallel those of haplogroup T5 – the rare subclade of T shared between European and Near Eastern breeds [9].

It is rather intriguing that until now all mtDNAs belonging to R have been detected in Italian breeds. This might be due to the over-representation of Italian cattle in our sample, but a survey of modern published datasets from other regions does not reveal mtDNA control regions compatible with this haplogroup status [16], [20]–[22]. If confirmed, our finding could indicate that there was something peculiar about Italy [5], [23], either in the *B. primigenius* stocks or in the Neolithic cattle breeding practice. Italy was one of the European refugia during the Last Glacial Maximum, but the post-glacial expansion of its refugial populations was restricted by the Alps to the North [24]. Thus, it is possible that some unique phenotypic features (e.g. size or behavior) of Italian wild aurochsen might have lessened the need by the first farmers and pastoralists to act against their admixture with domesticated stocks.

The low frequency of R (and P) mtDNAs in taurine cattle should not be underestimated. The matrilineal inheritance of mtDNA completely ignores the genetic input resulting from the interbreeding of aurochs bulls with domestic cows, thus it is likely that the overall extent of gene flow from European aurochs to modern cattle is higher than that revealed by mtDNA data alone. Unfortunately, Y-chromosome analyses have not yet provided definitive answers concerning this issue [19], [25], probably due to a still insufficient level of phylogenetic resolution.

In conclusion, modern taurine cattle breeds harbour at least two mtDNA haplogroups (R and P) that were transmitted by European aurochsen as the result of sporadic interbreeding events with domestic herds grazing in the wild. Whatever the reason and exact extent may be, the detection of R mtDNAs from European aurochsen in autochthonous Italian breeds, some of which are endangered because they are poorly specialized and economically marginal, identifies in such breeds an unexpected reservoir of genetic variation for modern cattle – variation previously believed to be lost and that we should now carefully preserve.

A third haplogroup, named Q, is also observed in modern taurine breeds from Italy. The ancestral homeland of this haplogroup is less clear. Most likely it represents an additional minor lineage that was domesticated in the Near East and later spread with human migrations and trades, but a definitive answer requires extensive coding-region data from a wide range of *B. taurus* populations, especially from the Near East. We advance the opinion that the mtDNA tree of cattle has eventually come of age [26] (similar to what happened to the human mtDNA tree almost a decade ago) and that future mtDNA studies need to follow complete genome analyses [8].

## Materials and Methods

### Samples

A total of 619 specimens from 22 Italian breeds and four breeds from other European regions were collected. DNAs were purified from either peripheral blood or platelets according to standard methods. All experimental procedures were reviewed and approved by the Animal Research Ethics Committee of the University of Pavia, in accordance with the European Union Directive 86/609.

### Sequences analysis of the mtDNA control region

For all animals, a PCR fragment of 1138 bp encompassing the entire mtDNA control region (15792-363) was sequenced from np ∼15805 to np ∼305 (about 840 bp), as previously described [9]. Sequences were aligned to the Bovine Reference Sequence (BRS) using the Sequencher software (Gene Codes Corporation) and the identified mutational motifs (Table 1) were used to classify mtDNAs within haplogroups.

### Sequencing of entire mitochondrial genomes

The entire mtDNA sequence of selected animals was obtained as previously reported [9]. In brief, a set of 11 overlapping PCR fragments covering the entire mtDNA genome was produced and sequenced by standard dideoxysequencing with 32 nested oligonucleotides. Raw sequence data were grouped into mtDNA genome contigs and compared to the BRS [13] to derive individual haplotypes.

### MtDNA phylogeny and time estimates

The tree was built and rooted by using a *Bos grunniens* (yak) and a *Bison bison* (American bison) mitochondrial genome, as previously described [9]. The evolutionary distances were computed using the Maximum Likelihood method together with averaged distance (ρ) of the haplotypes within a clade from the respective root haplotype, accompanied by a heuristic estimate of SE (σ). All positions containing gaps and ambiguous data were eliminated from the dataset. Estimate of the time to the most recent common ancestor for each cluster was calculated using a corrected age estimate of about 3,172 years per substitution in the whole coding region (15,428 bp) [9].

## Supporting Information

Table S1 Frequencies of mtDNA Haplogroups in Cattle Breeds
(0.06 MB DOC)
