# Supplementary material for: The Multifaceted Origin of Taurine Cattle Reflected by the Mitochondrial Genome
Source: PLoS One. 2009 Jun 1;4(6):e5753. doi: 10.1371/journal.pone.0005753 (PMC2684589; doi:10.1371/journal.pone.0005753)
Supplement: Table S1 — Frequencies of mtDNA Haplogroups in Cattle Breeds (0.06 MB DOC) [file pone.0005753.s001.doc]

**Supporting Information**

Table S1. Frequencies of mtDNA Haplogroups in Cattle Breeds

| Breeds^a^ | Haplogroups | | | | | | Total |
| --- | --- | --- | --- | --- | --- | --- | --- |
|  | T1’2’3 | T1 | T2 | T3 | Q | R |  |
| Agerolese | 1 | 4 | - | 27 | - | 1 | 33 |
| Bianca Val Padana | - | - | - | 4 | - | - | 4 |
| Italian Brown | - | 1 | - | 8 | - | - | 9 |
| Burlina | - | - | - | 1 | - | - | 1 |
| Cabannina | 1 | - | 1 | 25 | - | - | 27 |
| Calvana | - | 2 | - | 23 | - | - | 25 |
| Chianina | - | 5 | 1 | 43 | 1 | - | 50 |
| Cinisara | - | 5 | 1 | 21 | - | 2 | 29 |
| Garfagnina | - | - | 1 | 1 | - | - | 2 |
| Grey Alpine | - | - | - | 4 | - | - | 4 |
| Holstein | - | - | - | 2 | - | - | 2 |
| Italian Friesian | - | - | - | 11 | - | - | 11 |
| Italian Podolian | - | 8 | 3 | 65 | - | - | 76 |
| Italian Red Pied | - | 1 | 4 | 50 | 1 | - | 56 |
| Limousine | - | 3 | 1 | 46 | - | - | 50 |
| Maremmana | - | 3 | 3 | 16 | - | - | 22 |
| Modicana | - | - | - | 12 | - | - | 12 |
| Mucca Pisana | - | - | - | 20 | - | - | 20 |
| Ottonese | - | - | - | 7 | - | - | 7 |
| Piedmontese | - | - | 1 | 56 | - | - | 57 |
| Rendena | - | - | - | 1 | - | - | 1 |
| Romagnola | - | 1 | 5 | 41 | 2 | 1 | 50 |
| Savoiarda | - | - | - | 2 | - | - | 2 |
| Simmental | - | - | - | 9 | - | - | 9 |
| Swiss Brown | - | - | - | 1 | - | - | 1 |
| Valdostana | - | - | - | 2 | - | - | 2 |
| Unknown^b^ | - | - | 3 | 54 | - | - | 57 |
| *Total (%)* | *2 (0.3)* | *33 (5.3)* | *24 (3.8)* | *552 (89.2)* | *4 (0.7)* | *4 (0.7)* | *619* |

^a^ All listed animals belong to Italian breeds with the following exceptions: Limousine, Holstein, Simmental and Swiss Brown.

^b^ European cattle for which a specific breed affiliation was not available.
